# TRPV1: Structure, Endogenous Agonists, and Mechanisms

**DOI:** 10.3390/ijms21103421

**Published:** 2020-05-12

**Authors:** Miguel Benítez-Angeles, Sara Luz Morales-Lázaro, Emmanuel Juárez-González, Tamara Rosenbaum

**Affiliations:** Departamento de Neurociencia Cognitiva, División Neurociencias, Instituto de Fisiología Celular, Universidad Nacional Autónoma de México, Mexico City 04510, Mexico; mbenitez@ifc.unam.mx (M.B.-A.); saraluzm@ifc.unam.mx (S.L.M.-L.); ejuarez@ifc.unam.mx (E.J.-G.)

**Keywords:** TRP channels, TRPV1, pain, agonist, structure

## Abstract

The Transient Receptor Potential Vanilloid 1 (TRPV1) channel is a polymodal protein with functions widely linked to the generation of pain. Several agonists of exogenous and endogenous nature have been described for this ion channel. Nonetheless, detailed mechanisms and description of binding sites have been resolved only for a few endogenous agonists. This review focuses on summarizing discoveries made in this particular field of study and highlighting the fact that studying the molecular details of activation of the channel by different agonists can shed light on biophysical traits that had not been previously demonstrated.

## 1. Introduction

Since the Transient Receptor Potential Vanilloid 1 (TRPV1) ion channel was cloned in the last years of the 20th century [1], there have been extensive efforts to reveal the structural and functional details of this protein. Thus, nowadays it is one of the best characterized members of the TRP family of ion channels.

TRPV1 is mainly expressed by trigeminal ganglion (TG) neurons and by small-diameter neurons within sensory ganglia such as the dorsal root ganglion (DRG). It was first reported to be polymodal, responding to stimuli of different natures such as noxious heat (>43 °C) and capsaicin, the pungent compound present in hot chili peppers that functions as a chemical agonist of the channel, eliciting pain-associated behaviors in animals and pain in humans [2]. Soon after, it was shown that TRPV1 is also activated by low extracellular pH (pH ≤ 5.9). It is important to consider that when several stimuli are present together, TRPV1 activation is potentiated [2].

Several more agonists for this channel have been described and have been reviewed elsewhere [3,4,5,6,7,8,9,10,11]; however, this review will discuss some of the structural details of TRPV1 and focus only on those agonists of an endogenous nature that have been described for this channel.

## 2. Structural Overview of the TRPV1 Channel

The TRPV1 channel was not only the first mammalian TRP channel to be cloned [1] but it was also the first structure for a TRP channel to be resolved [12,13].

The first clues about TRPV1′s structure were obtained by relying on hydrophobicity analyses, suggesting that TRPV1 is a six-pass transmembrane protein (S1–S6) with a hydrophobic handle between S5 and S6 (pore region). The sequence alignment of the rodent TRPV1 protein showed that the long N-terminus contains multiple ankyrin repeats and a relatively short C-terminal region [1]. These intracellular domains are important scaffolds for interactions with other proteins and also contain binding sites for compounds that regulate TRPV1 function (i.e., calmodulin or CaM and ATP binding sites located at the N- and C- termini) [14,15,16].

Remarkably, capsaicin detection is abrogated in birds; although these animals also express TRPV1 channels, activation of these channels is dependent on protons but not on binding of the vanilloid compound [17]. Specific structural differences between avian and mammalian TRPV1 proteins result in dissimilar responses to capsaicin, since this compound binds to specific amino acids near the S3 of the mammalian TRPV1 protein and this vanilloid-binding pocket is different from that of the avian protein [17]. This finding was confirmed in 2015 [18], establishing that the capsaicin pocket is a hydrophobic cavity constituted by residues Y512, S513, T551, and E571 in the human TRPV1 sequence. While the first two residues are conserved between species, T551 is different in rabbit and chicken, the two species known to be insensitive to the pungency of chili peppers. Hence, these fine structural differences between TRPV1 orthologues produce channels with distinct susceptibilities to capsaicin [18].

Unlike the capsaicin pocket, the binding site for protons is conserved between species and it is located extracellularly at the S5 linker (E600 and E648). Interestingly, protons interact with the E600 amino acid residue and potentiate TRPV1 activation by other stimuli such as temperature or capsaicin, while E648 specifically regulates activation by protons [19]. Although the proton binding sites are conserved between species, a recent study showed that chicken TRPV1 activation by protons produces smaller currents than mouse TRPV1 [20]. Moreover, the avian channel is resistant to the typical desensitization produced by repeated application of TRPV1 agonists (i.e., protons) [20]. A structural determinant of TRPV1′s desensitization is the interaction of CaM at sites located at the N- and C-terminal regions [14,15]. Interestingly, it has been concluded that differences in the CaM binding site found in the C-terminus between these TRPV1 orthologues confer resistance to desensitization by protons in the avian channel [20].

Several lines of evidence have revealed binding sites for several TRPV1-activating compounds [2,21,22,23,24,25,26,27,28,29,30,31]. During the last two decades, the TRPV1 channel research field has produced a large body of evidence on the regulation of this channel by multiple compounds. Conversely, the determination of a high-resolution structure for TRPV1 was slower due to the experimental difficulties to crystallize this type of protein. The first contribution of this kind was the crystallization of the isolated ankyrin repeats and determination of their structure by X-ray diffraction methods [16]. This analysis solved the structure of the six ankyrin repeats of TRPV1 with a resolution of 2.7 and 3.2 Å and showed the typical 33-amino acid motifs forming antiparallel α-helices followed by a finger loop [23]. These motifs generate surfaces available for interactions with the ankyrins from other proteins. Additionally, the TRPV1 ankyrin repeats showed an electron density corresponding to an ATP molecule bound to these structures 1–3 [16], which has been shown to positively regulate TRPV1 activation.

Subsequently, the first 3D structure of the rat TRPV1 was determined by using electron cryomicroscopy (cryo-EM). The 19 Å, low-resolution structure of the full-length TRPV1 channel demonstrated the fourfold symmetry of this channel (similar to Kv channels), and the existence of two evident regions: the intracellular N- and C-terminal regions that resemble a basket domain and comprise ~70% of the total mass of the channel while the transmembrane region forms a compact and small domain [32]. Functional studies using fluorescence resonance energy transfer (FRET) showed that the N-termini surround the C-termini and the N-termini are farther away from the membrane than the C-termini [33].

Other findings related to the structural details of TRPV1 were mainly established by functional studies. For example, constrictions in the pore of TRPV1 were identified using an experimental strategy of accessibility to thiol-modifying agents [34] and important sites for the regulation of its gating properties were reported [35].

During the last years, our knowledge on the structure of TRPV1 was greatly enhanced by the obtainment of the first high-resolution structure (at a 3.4 Å resolution) through single-particle cryo-electron microscopy (cryo-EM) [12]. This TRPV1 structure showed the classic fourfold symmetry of this channel, and clearly resolved the transmembrane helices S1–S6, the TRP box, and the re-entrant loop between S5 and S6, corresponding to the pore region. Intracellular domains such as the N-terminal ankyrin repeats were also identified and the TRP box at the C-terminus was shown to be oriented towards the S4–S5 linker and the pre-helix-S1, suggesting an essential role for it in the allosteric modulation of TRPV1 channels [12]. The outer pore region resembles a wide funnel structure and the small selectivity filter was observed further down the channel and shown to contain a signature sequence (643-GMGD-646). The cryo-EM TRPV1 structure also showed a deep constriction site, near a previously suggested gate [34].

Several TRPV1 structures were obtained in these experiments. An “apo-state” of the channel was determined in the absence of agonist [34]. In addition to the “apo”-TRPV1 structure, there are two additional structures: one in the presence of agonists resiniferatoxin (RTX) together with the double-knot toxin (DkTx) and another in the presence of capsaicin [13]. RTX is a compound found in the “cactus-like” plant *Euphorbia resinifera* that functions as an agonist of TRPV1 by binding to the vanilloid pocket of the channel but that is more potent than capsaicin [11,36,37,38]. On the other hand, DkTx is a peptide toxin found in the venom of the *Ornithoctonus huwena* spider. This peptide contains two inhibitor-cysteine-knots (ICK) motifs called K1 and K2 lobes which interact with TRPV1 through several external aromatic residues [30]. The toxin stretches out into a space normally filled by lipids in the absence of the toxin constituted by S4, S6, and the pore helix, where it interacts with the lipid membrane [39] and gates the channel through a mechanism different to that of capsaicin [40].

The 3D structure with DkTx and RTX unveiled the full-open TRPV1 channel with a resolution of 3.8 Å. This reconstruction revealed that DkTx binds to the extracellular loops of the tetrameric channel and shows an electronic density in the vanilloid pocket suggesting that RTX is situated at this binding site [1]. Similarly, a weaker electronic density at the vanilloid pocket was observed in the reconstruction of the 3D TRPV1 structure with capsaicin (resolution at 4.2 Å), perhaps reflecting the fact that capsaicin displays less affinity to this site than RTX. Interestingly, these densities are in close proximity, although there is not a complete overlap, which suggests that these agonists bind to the same pocket but they do not interact with the same amino acids [13].

Ligand-bound TRPV1 structures provided information on the existence of different pore profiles in this channel. Firstly, the capsaicin-bound structures displayed clear expansion at the lower gate in comparison to the “apo”-state. Moreover, the full-open channel structure (DkTx/RTX-TRPV1) revealed that the ion pore conduction lacks any structural constriction [13]. The outer pore region is also rearranged in the full-open channel since a substantial change at the position of the helix pore confers inflexibility to this region. Consequently, the helix moves away from the central axis and transduces a conformational change at the loop between S5 and S6. This loop contains two glutamic acids (E600 and E648) which are essential to TRPV1 activation/potentiation by protons. The apo-TRPV1 state shows interaction between E600 and two contiguous amino acids (Y663 and D654), maintaining the channel in an inactivated state. However, the interaction of these amino acids is interrupted in the DkTx/RTX-TRPV1 structure, which results in an increase in the distance between these amino acids and maintains the channel in a fully conducting form [13]. These changes at the pore suggest high flexibility of this region and provide evidence to the hypothesis that each agonist can lead to a different gating mechanism. Finally, three high-resolution TRPV1 structures have been recently obtained combining the cryo-EM with the lipid nanodisc technology. These structures correspond to the apo channel and agonist- and antagonist-bound states [41].

To obtain these better resolution TRPV1 structures, the channels were embedded in a lipid bilayer-like environment provided by the, promoting a structural arrangement closer to the native environment and yielding density maps of higher qualities. In these nanodisc structures, the electronic densities of lipids interacting with the channel were well resolved and a tripartite complex of lipids–TRPV1–toxin was obtained at a 2.9 Å resolution. The elucidation of this structure determined that the two hydrophobic fingers of DkTx are inserted into the bilayer, where the aliphatic tail of a phospholipid interacts with the side chain of the W11 residue of finger 1 of DkTx and the negatively charged head of this lipid electrostatically interacts with residue R534 located at the S3-S4 extracellular loop [41].

In addition, hydrophobic interactions were shown to be established between a phenylalanine located in finger 2 of the toxin and the aliphatic tail of the lipid while the head group is coordinated by extracellular residues located at the pore helix (S629). These data suggest that the toxin induces molecular arrangements in TRPV1 in a phospholipid-dependent manner stabilizing the full-activated state of the channel [41].

Strikingly, the TRPV1 apo-state obtained at 3.2 Å resolution showed a phosphatidylinositol lipid associated to the vanilloid pocket, where the acyl chain is lengthened along S4 of one subunit and towards S5-S6 of the adjacent subunit. This lipid is displaced from the vanilloid pocket in the TRPV1 structure associated to RTX and this binding pocket is also occupied by capsazepine, a TRPV1 antagonist [41].

Since several of the endogenously produced molecules that will be here discussed have been shown or proposed to interact with the vanilloid-binding pocket, we will next describe how vanilloid molecules were shown to interact at this site in the cryo-EM structures. As mentioned above, the structure obtained in the presence of capsaicin alone was considered a “partially open” structure because the presence of this agonist only produced the opening of the lower gate and no changes at the region of the selectivity filter were evident in the presence of capsaicin [13]. The cryo-EM structures also confirmed previous mutagenesis studies that showed that residues Y511 (whose aromatic ring points toward the pocket and attracts the ligands), S512 in the S3, and M547 and T550 in the S4 are all important for vanilloid binding and that capsaicin and/or RTX are coordinated in a pocket above residue E570 in the S4-S5 linker of one subunit [17,42,43]. This E570 residue is proximal to residue L669 in the S6 of the neighboring subunits; hence, this vanilloid binding site could putatively affect gating by impacting on both regions, the S4-S5 linker and the S6. In summary, the idea is that the RTX or capsaicin can interact with residues in the S4-S5 linker and pull them away from the central pore, leading to the opening of the channel [12,13,41,44].

More recently, details on the stoichiometry of TRPV1 activation by capsaicin were provided by Liu and collaborators [45]. These authors used microfluorometry to measure intracellular Ca^2+^ increases in HEK293 cells that expressed linked tetrameric TRPV1 receptors with different subunit compositions that contained the capsaicin-insensitive S512F mutant and/or wild-type subunits. The authors covalently linked the subunits in order to produce all possible tetrameric compositions containing one single wild-type repeat and observed that the mutant channels could be partially opened by capsaicin and that the binding of two vanilloid molecules (i.e., two wild-type and two mutant subunits) is required in order to fully transduce capsaicin-dependent stimuli [45].

**Figure 1:**
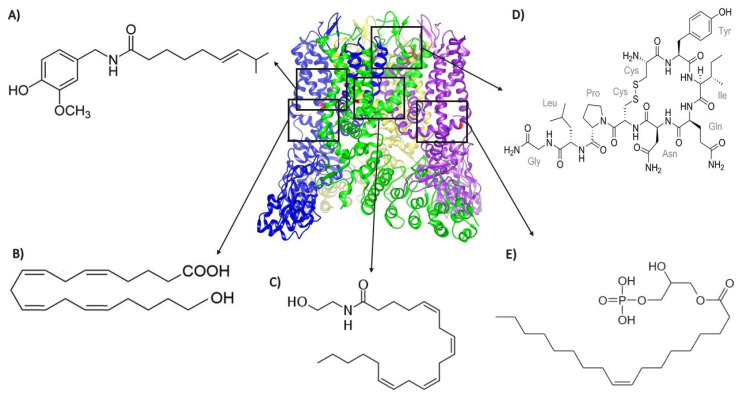
Structure of TRPV1 depicted with different agonists bound to various sites in the protein. Except for (**A**) capsaicin (that binds to residues Y512, S513, T551, and E571), all other agonists are endogenously produced: (**B**) 20-hydroxyeicosatetraenoic acid (20-HETE, which interacts with residue S502); (**C**) anandamide (which interacts with residues Y511, S512, and R591); (**D**) oxytocin (which interacts with residues E600, G602, Y631, and L635); and (**E**) lysophosphatidic acid (LPA, which binds to the K710 residue). The 3j5q PDB file that corresponds to the open structure (obtained with RTX and DkTx) of TRPV1 [41] was used. The black squares represent the regions of the channel where the different depicted endogenous agonists bind.

In summary, the last years of the TRPV1 research field have seen growth, enhanced by the pivotal information provided by high-resolution structures and along the way establishing cryo-EM as the best tool for structural analysis of several members of the TRP channel family. As will be detailed below, the last decade has also witnessed the discovery of several endogenously produced agonists (molecules listed in Table 1) of this channel, albeit interaction sites with the protein have been deciphered only for some of these molecules (Figure 1).

## 3. Endogenously Produced Agonists of TRPV1

### 3.1. Products Derived from Polyunsaturated Fatty Acids

Fatty acids are large chains of monocarboxylic acids (8 to 22 carbons), the synthesis of which occurs through the successive addition of acetyl CoA. In mammals, fatty acids are found in their saturated form [46] while polyunsaturated fatty acids (PUFAs) are provided through diet.

PUFAs are classified into two families: n-3 or ω-3 (α-linolenic acid or ALA; docosahexaenoic acid or DHA; eicosapentaenoic acid or EPA; also see Table 1 with abbreviations for all compounds mentioned) and n-6 or ω-6 (arachidonic acid, linoleic acid, γ-linolenic acid), according to the position of the first double bond present in their structures [47,48].

**Figure 2:**
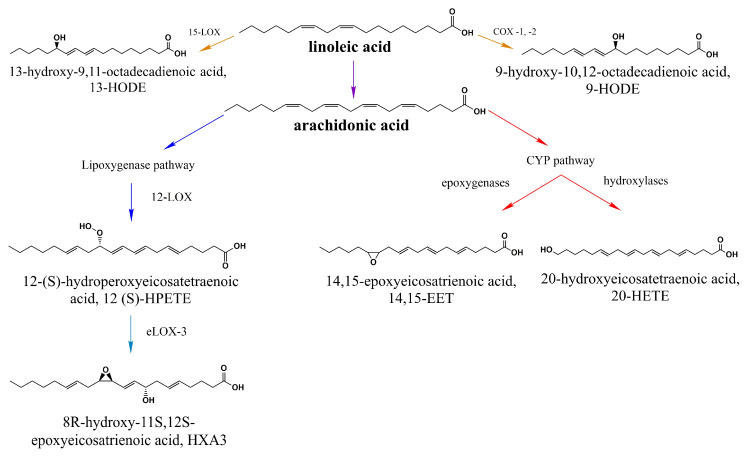
Products of linoleic acid (LA) that activate TRPV1. LA is the precursor of several long-chain polyunsaturated fatty acids including arachidonic acid, 9-HODE, and 13-HODE. All byproducts downstream of the lipooxygenase and cytochrome P450 (CYP450) pathway shown in this scheme have been proposed to activate TRPV1 [51,52].

In the body, specifically in the hepatocyte’s endoplasmic reticulum, they are transformed (elongated and desaturated) and form long-chain polyunsaturated fatty acids, starting from reactions orchestrated by malonyl CoA. In this way, linoleic acid (LA 18:2) serves as a precursor to several other molecules such as arachidonic acid (AA 20:4) [47]. Then, this is followed by hydroxylation or epoxidation reactions catalyzed by cytochrome P450 (CYP450) enzymes, resulting in the generation of hydroxyeicosatetraenoic acids (HETEs), such as 20-hydroxyeicosatetraenoic acid (20-HETE), or epoxieicosatrienoic acids (EETs) [49,50] (Figure 2).

**Figure 3:**
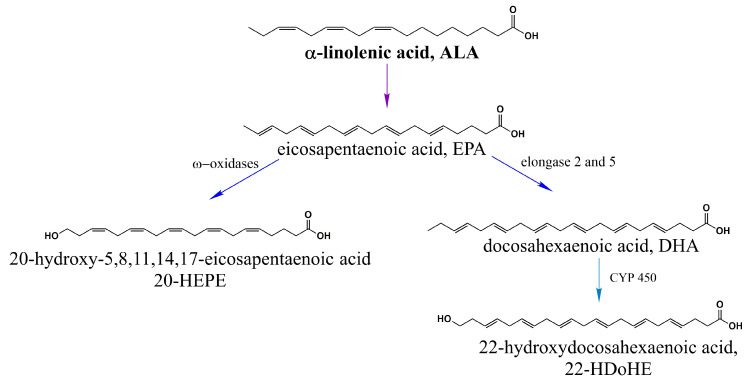
α-linolenic acid (ALA, n-3) activates TRPV1. ALA is a precursor of EPA and EPA is transformed into 20-HEPE via ω-oxidation and into DHA through elongation reactions. 22-HDoHE is a polyunsaturated fatty acid which is derived from DHA through a ω-hydroxylation reaction catalyzed by the cytochrome P450 enzyme omega-hydroxylase.

α-linolenic acid (ALA 18:3) produces eicosapentaenoic acid (EPA 20:5) and docosahexaenoic acid (DHA 22:6) [47], which are precursors of 20-hydroxyeicosapentaenoic acid (20-HEPE) and 22-hydroxyeicosapentaenoic acid (22-HDoHE) [50] (Figure 3).

In 2000, Hwang and collaborators proved that hydroxyeicosapentaenoic acid (12 (S)-HPETE, the (S)-enantiomer of 12-HPETE derived from AA, Figure 2) is capable of activating the TRPV1 channel [53] and this discovery led to further studies which investigated the effects of other PUFA-derived molecules on the activation of TRPV1, as mentioned below.

It has also been shown that hepoxylin (HXA3 and HXB3; Figure 2), a product of arachidonic acid and 12(S)-HPETE, has the ability of triggering Ca^2+^ mobilization in a heterologous expression system (HEK cells) that stably expresses the TRPV1 and Transient Receptor Potential Ankyrin 1 (TRPA1) channels as well as in rat sensory neurons. It is worth mentioning that TRPA1 channels are also noxious stimuli-sensing proteins that are coexpressed with TRPV1 in some sensory neuron subpopulations, hence the importance of evaluating the effects of the above-mentioned agonists on the activity of both channels [54]. Notably, when applied to cells from both TRPV1 and TRPA1 knock-out (KO) animals or when antagonists of these channels (AMG9810 for TRPV1 or HC030031 for TRPA1) are applied, these effects are attenuated. Thus, these results lead to the conclusion that HXA3 promotes tactile allodynia and hyperalgesia mediated by the activation of TRPV1 and TRPA1 channels [55].

20-HETE, which is also derived from AA, is capable of activating and sensitizing TRPV1 in humans and in mice [22]. According to Wen and collaborators, the activation might imply the direct binding of 20-HETE to residue S502 or conversely, considering that the site is fundamental for functional phosphorylation of TRPV1 [56,57], it could promote activation through mechanisms involving protein kinase A (PKA, which is dependent on cyclic adenosine monophosphate or cAMP) and/or protein kinase C (PKC, which is dependent on Ca^2+^). This, in turn, would lead to the opening of the channel in the absence of the direct binding of 20-HETE. Interestingly, it has also been reported that channel opening and its sensitization occur through actions of 20-HETE on different sites; while mutation of S502 affected only the opening of the channel, it did not affect its sensitization or its activation by capsaicin [22].

Other PUFAs such as 20-hydroxyeicosapentaenoic acid (20-HEPE, derived from EPA) and 22-hydroxydocosahexaenoic acid (22-HDoHE, derived from DHA) have been shown to be more efficient than 20-HETE for TRPV1 activation while expressed in HEK cells but they did not produce pain in a murine model [50]. Hwang et al. hypothesized that, since both molecules belong to the ω-3 family of fatty acids, due to their intrinsic structural features, these could either interact with a different binding site on the channel or bind with higher affinity than 20-HETE [50].

Since the beginning of the decade, 9- and 13-hydroxyoctadecadienoic acids (9-HODE and 13-HODE, derivatives of LA) were proposed as agonists of the TRPV1 channel. The presence of both molecules was detected in depolarized spinal cord neurons in rats [51], in skin biopsies of mice and rats when they were exposed to harmful heat >43 °C [52], and when LA was exogenously applied to DRG neurons [58].

In endogenous and heterologous expression systems, TRPV1 is activated by 9- and 13-HODE, as well as by its oxidized forms 9- and 13-oxoODE, causing mechanical allodynia [51], heat sensitivity in rodents [52], and inflammatory hyperalgesia induced by λ-carrageenan [58], a soluble polysaccharide isolated from sea plants that causes inflammation and pain [59].

The latter effects were not reproducible when neurons from TRPV1 KO mice were used or when the heterologous system was exposed to TRPV1 antagonists (i.e., I-RTX and capsazepine) or to PD146176, a 15-lipoxygenase blocker (enzyme responsible for metabolizing LA to HODEs, mainly 13-HODE; [60]), or when they were immunoneutralized [51,52,58].

Although a detailed mechanism for TRPV1′s direct activation by byproducts of LA has not been established, these reports suggest that this is a possibility. As mentioned by Patwardhan and collaborators in their 2009 study, several metabolites of linoleic acid could be contributing to the activation of the channel and acting in concert (entourage effect), as seen for other lipid families [51].

It has been shown that mutants of TRPV1 at positions 511 and 512, that are characterized by being able to respond to heat and pH but not to capsaicin, did exhibit Ca^2+^ mobilization in the presence of 9-HODE, suggesting that the binding site for this compound is different from that of capsaicin [52].

In 2016, a lipidomic profile of the rat’s spinal cord after burn injury was determined for the first time. In this study, abnormal levels of HODEs were not observed, although an increase in hydroxy- and epoxy- metabolites of linoleic acid, namely, 9,10-dihydroxy-12Z-octadecenoic acid (9,10-DiHOME); 12,13-dihydroxy-9Z-octadecenoic acid (12,13-DiHOME); 9(10)-epoxy-12Z-octadecenoic acid (9(10)-EpOME); and 12(13)-epoxy-9Z-octadecenoic acid (12(13)-EpOME), was reported in spinal cord tissue [61].

Then, activation of several TRP channels by 9,10-DiHOME, 12,13-DiHOME, 9(10)-EpOME, and 12(13)-EpOME was assessed using the patch-clamp technique and it was shown that these metabolites activated TRPV1 and TRPA1 but not TRPV2, TRPV3, TRPV4, or Transient Receptor Potential Melastatin 8 (TRPM8, a cold-sensing ion channel that also responds to compounds such as menthol and icilin) [61,62].

Moreover, in vivo experiments showed that four LA metabolites applied to the spinal cord induced allodynia, an effect that was partially blocked with the TRPV1 antagonist, AMG9810, or with HC030031 (a TRPA1 antagonist). Furthermore, allodynia produced by these LA metabolites was completely eliminated by using both antagonists at the same time [61].

### 3.2. Endocannabinoids

Endocannabinoids are endogenous ligands that activate CB1 and CB2 cannabinoid receptors and are derived from esters, ethers, and amides of PUFAs such as arachidonic acid [63]. Nonetheless, these molecules possess a chemical structure that allows many of them to activate other types of proteins, including TRPV1 [64].

#### 3.2.1. N-Acyl Amides

N-acyl amides are one of the main groups of simple lipids with structures consisting of a fatty acid (acyl group) attached to a simple amine by an amide link where amines can be substituted by amino acids or their byproducts, if they can be conjugated with a fatty acid [65].

N-acyl amides are considered “orphan lipids”, since no specific receptor for these molecules has been discovered; nevertheless, there is evidence of their ability to bind to different endocannabinoid receptors including CB1 and CB2, to G-protein-coupled receptors (GPCRs) 18 and 55 (GPR18 and 55), and to some members of the TRP ion channel family [65].

**Figure 4:**
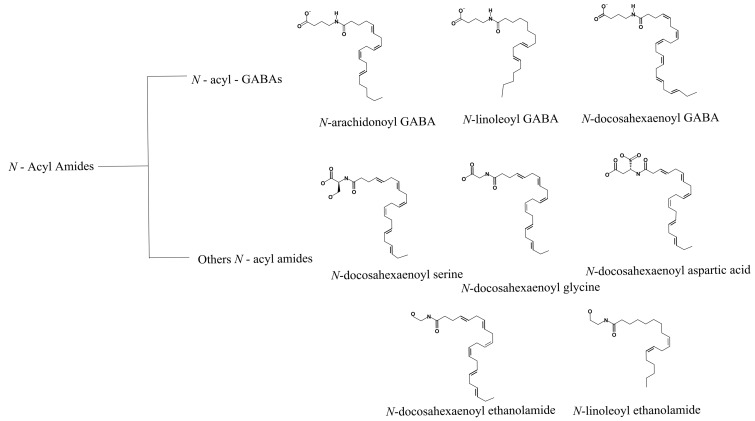
N-acyl amides with agonist effects on TRPV1. The family of N-acyl GABA molecules that are structurally related is shown in the top panel. The middle and lower panels depict the structures of other N-acyl amides that arise from different families and that do not necessarily share functional groups but also activate TRPV1.

In 2014, Bradshaw and collaborators reported different N-acyl amides as activators of TRP ion channels (TRPV1-V4). Using an internal library of 70 N-acyl amides and measurements of changes in intracellular Ca^2+^ levels through fluorescent ratiometric indicators (i.e., Fura-2 AM), these authors identified several agonists of the TRPV1 channel [66] (Figure 4).

Other molecules such as N-acyl GABA and similar compounds such as N-docosahexaenoyl GABA (D-GABA), N-linoleoyl GABA (L-GABA), and N-arachidonoyl GABA (A-GABA) also resulted in Ca^2+^ mobilization into the cell in a concentration-dependent fashion. In addition to N-acyl GABA and its derivatives, five other N-acyl amides that were more efficient in activating TRPV1 were identified: N-docosahexaenoyl ethanoyl serine, N-docosahexaenoyl glycine, N-docosahexaenoyl aspartic acid, N-docosahexaenoyl ethanolamide, and N-linoleoyl ethanolamide [66] (Figure 4). In summary, by testing several biologically active lipids, the authors concluded that long-chain unsaturated acyl amides function as TRPV1 activators. Nonetheless, work still needs to be performed to determine the mechanisms by which these molecules modulate the function of TRPV1 and establish binding sites for these in the ion channel.

#### 3.2.2. N-Acylethanolamines

N-Acylethanolamines *(NAEs)* are lipids which, in most cases, originate as products of N-acyl-phosphatidylethanolamine (NAPE) hydrolysis by the specific D-NAPE phospholipase [67]. They are characterized by the presence of a variable length fatty acid chain linked to an ethanolamine through an amine and are classified based on the number of carbons they possess and on the level of saturation of the acyl chain [68].

Anandamide (AEA), an endocannabinoid and an NAE, was the first endogenous agonist described for TRPV1 and it is characterized by also binding to the vanilloid pocket [69,70], as capsaicin does. Moreover, several studies have revealed that AEA’s production is accompanied by the generation of other NAEs, particularly palmitoylethanolamide PEA (C16:0) and oleoylethanolamine OEA (C18:1) [71].

NAEs have been shown to be effectors of TRPV1 activity (Figure 4). For example, N-oleoyl ethanolamine (18:1 NOE or OEA) gives rise to TRPV1 currents in cells previously sensitized with PKC [30,31] and intraperitoneal OEA administration leads to visceral pain behavior, which is not observed in TRPV1-null mice [72], supporting a nociceptive role for OEA through the activation of TRPV1 ion channels [73].

PEA was shown to be present in egg yolk, soybean lecithin, and peanut oil since the 1950s [74] and has since been described to exhibit analgesic and anti-inflammatory properties [75]. Structurally, it is similar to anandamide and other endocannabinoids and shares the same synthesis and degradation routes as these [76].

Despite its classification as an endocannabinoid, PEA is weakly linked to the function of the CB1 and CB2 receptors [69]; however, in the last decade, it has been suggested that it interacts with other receptors such as PPARα (Peroxisome Proliferator-Activated Receptor alpha) and TRPV1 [77,78].

In 2008, a link between TRPV1 and PEA was suggested when it was shown that the application of PEA to the sciatic nerve of rats had an antihyperalgesic effect, which was partially reduced with capsazepine [79]. Other experiments demonstrated that PEA induced dose-dependent vasodilation of endothelial mesenteric arteries (and that it also potentiated the effect of AEA, although PEA is even less potent than OEA at activating TRPV1), a phenomenon which was absent when TRPV1 was inhibited with the antagonist SB36679 [80,81]. In synthesis, these experiments suggested a possible interaction between PEA and TRPV1.

A few years later, PEA was proposed to activate TRPV1 in a somatic cell hybrid cell line composed of rat embryonic DRG neurons with a mouse neuroblastoma cell line (F11) since it was shown to increase intracellular Ca^2+^ concentrations in a dose-dependent fashion that was blunted if TRPV1 antagonists such as capsazepine and SB36679 were applied. The authors suggested that this activation occurred through a mechanism that involved PPARα, since other experiments showed that PEA-induced TRPV1 currents were inhibited by 50% with GW-6471, a PPARα antagonist, an effect that was not observed when TRPV1 was activated by capsaicin [82].

Nonetheless, in contrast with the mechanism proposed above, it was also found that PEA activated transiently transfected TRPV1 in CHO (Chinese hamster ovary) cells, where patch-clamp experiments suggested that TRPV1 was activated and desensitized by PEA in a similar way to what occurs with capsaicin [82].

Finally, the synergistic effects of PEA and tramadol (an opioid analgesic) on pain-like behavior have also been assessed using the formalin test. Experiments have shown that a more efficient effect on analgesia can be achieved for PEA if it is used together with tramadol and that this effect depends upon the activities of the CBRs, TRPV1, and the PPARα [83].

### 3.3. N-acyl Amino Acids/Neurotransmitters

N-acyl amino acids (NAANs) are lipidic compounds with different headgroup moieties (such as glycine, dopamine, or GABA) that are conjugated to long-chain fatty acids and which can potentially participate in the modulation of the function of membrane proteins such as GPCRs, ion channels, and transporters. More than 70 endogenous NAANs with potential roles in the vasculature and the nervous and immune systems have been identified [84].

Recently, the presence of TRPV1 in the soma of pyramidal neurons of the prelimbic cortex of the prefrontal cortex (PFC) has been reported [85]. This finding is interesting since the activation of TRPV1 produces an increase in the frequency of spontaneous excitatory postsynaptic currents (sEPSC) in the substantia gelatinosa of the rat spinal cord and in the substantia nigra [86,87].

It has been shown that TRPV1 is activated by N-arachidonoyl taurine (NAT) [88]. Moreover, it has been demonstrated that when capsaicin and NAT activate TRPV1, the compounds do not modify the amplitude of the sEPSC but rather increase their frequency by 150–175% (as compared to the control group) before desensitization occurs, effects that are prevented when capsazepine is applied [85]. Hence, these data suggested a role for TRPV1′s activity in the generation of sEPSC and in modifying the excitatory glutamatergic transmission.

### 3.4. Oxytocin

Oxytocin is a hormone that is produced in the paraventricular and supraoptic nuclei of the hypothalamic nucleus, as well as in the intermediate accessory nuclei [89], and that stimulates the contraction of the uterine smooth muscle and the induction of lactancy, and regulates complex social behavior [90] by binding to its canonical G-protein-coupled oxytocin receptor (OXTR).

Recently, by using the PLC inhibitor U773122, it has been shown that oxytocin activates TRPV1 independently of the OXTR and that it induces the entrance of Ca^2+^ in a heterologous TRPV1 expression system and in F11 cells as well as in artificial bilayers. The direct effect of oxytocin on TRPV1 was confirmed with knock-out mice where the activity of the hormone is null. Computational predictions were performed to obtain specific mutants, which were experimentally tested to show that oxytocin interacts with TRPV1 through L635 and F649, suppressing pain by the activation and then desensitization of the channel [91].

### 3.5. Hydrogen Sulfide

Hydrogen sulfide (H_2_S) is an endogenous gaseous molecule that is a subproduct of cystathionine β-synthase (CBS) and cystathionine γ-lyase (CSE), which use L-cysteine as their main substrate. H_2_S functions as an important cytoprotective and exhibits antioxidant, anti-inflammatory, antiapoptotic, and muscle relaxant properties.

Since the middle of the last decade, it was described that H_2_S affects TRPV1′s activity and this, in turn, has consequences on the function of certain organs leading to contraction of the urinary bladder of rats and of the respiratory tract of guinea pigs [29,92] as well as affecting secretion from the colon of guinea pigs and humans through the local release of substance P after activation of TRPV1 [93].

Sodium hydrosulfide (NaHS), a product of half-neutralization of H_2_S with sodium hydroxide, induces a biphasic effect on the spontaneous contraction of the duodenal longitudinal muscle strips of rats. Specifically, at 1–2 min after NaHS (1 mM) administration, there was an increase in the contractility of the muscle (excitatory effect) that returned to basal levels within 5 min and, following this excitatory effect, there was a long-lasting (more than 30 min) inhibition of the contractility that depended on the actions of TRPV1 present in afferent nerve fibers, the tachykinin receptor 1 (TACR1), also known as neurokinin 1 (NK1) receptor, and of potassium channels from the smooth muscle [94].

Another study showed that NaHS promotes gastric acid secretion which is attenuated by antagonists of TRPV1 (i.e., capsazepine), of the NK1 receptor (i.e., L703606), and of the nuclear factor NF-κB (i.e., pyrrolidine dithiocarbamate), suggesting that activation of TRPV1 channels from the sensory nerve endings by H_2_S is partly responsible for this phenomenon [95].

Both of the studies mentioned above proposed that H_2_S activates TRPV1 channels from sensory nerve endings promoting the release of substance P (an undecapeptide that functions as a neurotransmitter and a modulator of pain perception by altering cellular signaling pathways) and subsequent activation of its NK1 receptor, that is expressed in a cell-specific manner in the digestive system such as in the smooth muscle, interstitial cells of Cajal, the endothelium of blood vessels, white blood cells, fibroblasts, and neurons [96,97].

### 3.6. Glycerophospholipids

Up to here we have discussed that TRPV1 is an ion channel that is activated by several agonists and that it undergoes characteristic structural changes in the presence of different activators. In this regard, it is worthwhile to note that TRPV1 is also activated by some glycerophospholipids.

Our group has previously reported that TRPV1 directly interacts with lysophosphatidic acid (LPA, [31]). LPA, similarly to TRPV1, has been extensively linked to the generation of chronic neuropathic pain. For several years, it was proposed that this phenomenon was only due to the interaction of LPA with its specific GPCRs [98,99,100,101], modulating the activity of several ion channels [5,102,103,104]. Most effects of LPA on other channels are related to how it regulates the expression levels of these proteins [105] and reports of direct interactions of LPA with ion channels in general are limited [104,106,107].

We have previously shown that LPA produces acute pain in mice and that, in HEK293 cells transfected with TRPV1, currents could be induced when LPA was applied to membrane patches expressing the ion channel and identified a site of interaction for this phospholipid, residue K710 in the C-terminus of TRPV1 [31]; this was later confirmed by another group in a study where TRPV1 was expressed in lipid bilayers and activated by LPA [108]. We also showed that activation of TRPV1 by compounds similar in structure to LPA was sensitive to the presence of a single unsaturation, an aliphatic chain of at least 18 carbons and the presence of a negatively charged group (i.e., phosphate); other combinations of these features failed to effectively activate the channel [109].

In a more recent study, we emphasized the biophysical details with which LPA promotes larger macroscopic currents than those promoted by saturating capsaicin concentrations [110]. However, this result could be explained by several mechanisms that included LPA producing an increase in the number of ion channels in the membranes of cells and an increase in the membrane negative surface charge resulting in the accumulation of positively charged ions near the pore mouth of TRPV1, a phenomenon termed “pore dilation” in which the permeability to large ions occurs in the presence of prolonged exposures to agonists [111] and/or a change in the single-channel conductance.

By performing several experiments, we ruled out all of the first three possibilities and showed that LPA could produce an increase in the single-channel conductance and that this phenomenon depended upon the presence of the K710 residue in the TRP box of TRPV1 [110].

## 4. Conclusions

TRPV1 is a polymodal protein with several important functions that include its role in nociception. This ion channel has proven to be an extraordinary example of functional and structural flexibility. Several agonists have been identified for TRPV1 and the presence and activity of this ion channel have been described in several organs. In this review, we have summarized findings related to new endogenous agonists for TRPV1 and the resolution of the fine details of its structure. It is clear that more work is still needed because, for several agonists, fine mechanisms of action have not yet been meticulously clarified. An example of what we can learn from studying how agonists affect TRPV1′s function is that we found that LPA, a molecule that exhibits increased levels during certain inflammation processes and diseases, is widely associated with pain generation, and that binds to the C-terminus of TRPV1, induces a proposed different conformational open state with a distinct single-channel conductance to that obtained in the presence of capsaicin. This larger single-channel conductance results in larger macroscopic currents in comparison to those produced by saturating concentrations of capsaicin and this could be physiologically relevant because it would enable the neurons that express this channel to depolarize more efficiently during certain pathophysiological situations.

## Figures and Tables

**Table 1:** Abbreviations for compounds.

Abbreviation	Name
AA	Arachidonic acid
AEA	Anandamide
A-GABA	*N*-arachidonoyl GABA
ALA	α-Linolenic acid
COX-1,-2	Cyclooxygenase-1,-2
CYP450	Cytochrome P450
D-GABA	*N*-docosahexaenoyl GABA
DHA	Docosahexaenoic acid
EETs	Epoxyeicosatrienoic acids
eLOX-3	Epidermis-type lipoxygenase 3
EPA	Eicosapentaenoic acid
HETEs	Hydroxyeicosatetraenoic acids
HXA3	Hepoxilin A3
HXB3	Hepoxilin B3
H_2_S	Hydrogen sulfide
LA	Linoleic acid
L-GABA	*N*-linoleoyl GABA
LPA	Lysophosphatidic acid
NAANs	*N*-acyl amino acids/neurotransmitters
NAEs	*N*-acylethanolamines
NaHS	Sodium hydrosulfide
NAPEs	N-acylphosphatidylethanolamines
OEA or NOE	Oleoyl-ethanolamine
PEA	Palmitoylethanolamide
PUFAs	Polyunsaturated fatty acids
9-HODE	9-hydroxy-10E,12Z-octadecadienoic acid
9-oxoODE	9-oxo-10E,12Z-octadecadienoic acid
9, 10-DiHOME	9,10-dihydroxy-12Z-octadecenoic acid
9(10)-EpOME	9(10)-epoxy-12Z-octadecenoic acid
12(S)-HPETE	12(S)-hydroperoxyeicosatetraenoic acid
12, 13-DiHOME	12,13-dihydroxy-9Z-octadecenoic acid
12(13)-EpOME	12(13)-epoxy-9Z-octadecenoic acid
12/15 -LOX	12/15-lipoxygenase
13-HODE	13-hydroxy-9Z, 11E-octadecadienoic acid
13-oxoODE	13-oxo-9Z,11E-octadecadienoic acid
20-HEPE	20-hydroxyeicosapentaenoic acid
20-HETE	20-hydroxyeicosatetraenoic acid
22-HDoHE	22-hydroxydocosahexaenoic acid

## References

[1] Caterina M.J., Schumacher M.A., Tominaga M., Rosen T.A., Levine J.D., Julius D. (1997). The capsaicin receptor: A heat-activated ion channel in the pain pathway. Nature.

[2] Tominaga M., Caterina M.J., Malmberg A.B., Rosen T.A., Gilbert H., Skinner K., Raumann B.E., Basbaum A.I., Julius D. (1998). The cloned capsaicin receptor integrates multiple pain-producing stimuli. Neuron.

[3] Morales-Lázaro S.L., Simon S.A., Rosenbaum T. (2013). The role of endogenous molecules in modulating pain through transient receptor potential vanilloid 1 (TRPV1). J. Physiol. (Lond.).

[4] Morales-Lázaro S.L., Lemus L., Rosenbaum T. (2017). Regulation of thermoTRPs by lipids. Temperature (Austin).

[5] Hernández-Araiza I., Morales-Lázaro S.L., Canul-Sánchez J.A., Islas L.D., Rosenbaum T. (2018). Role of lysophosphatidic acid in ion channel function and disease. J. Neurophysiol..

[6] Carnevale V., Rohacs T. (2016). TRPV1: A target for rational drug design. Pharmaceuticals.

[7] Siemens J., Zhou S., Piskorowski R., Nikai T., Lumpkin E.A., Basbaum A.I., King D., Julius D. (2006). Spider toxins activate the capsaicin receptor to produce inflammatory pain. Nature.

[8] Yang S., Yang F., Wei N., Hong J., Li B., Luo L., Rong M., Yarov-Yarovoy V., Zheng J., Wang K. (2015). A pain-inducing centipede toxin targets the heat activation machinery of nociceptor TRPV1. Nat. Commun..

[9] Starowicz K., Nigam S., Di Marzo V. (2007). Biochemistry and pharmacology of endovanilloids. Pharmacol. Ther..

[10] Szallasi A., Cortright D.N., Blum C.A., Eid S.R. (2007). The vanilloid receptor TRPV1: 10 years from channel cloning to antagonist proof-of-concept. Nat. Rev. Drug Discov..

[11] Elokely K., Velisetty P., Delemotte L., Palovcak E., Klein M.L., Rohacs T., Carnevale V. (2016). Understanding TRPV1 activation by ligands: Insights from the binding modes of capsaicin and resiniferatoxin. Proc. Natl. Acad. Sci. USA.

[12] Liao M., Cao E., Julius D., Cheng Y. (2013). Structure of the TRPV1 ion channel determined by electron cryo-microscopy. Nature.

[13] Cao E., Liao M., Cheng Y., Julius D. (2013). TRPV1 structures in distinct conformations reveal activation mechanisms. Nature.

[14] Numazaki M., Tominaga T., Takeuchi K., Murayama N., Toyooka H., Tominaga M. (2003). Structural determinant of TRPV1 desensitization interacts with calmodulin. Proc. Natl. Acad. Sci. USA.

[15] Rosenbaum T., Gordon-Shaag A., Munari M., Gordon S.E. (2004). Ca^2+^/calmodulin modulates TRPV1 activation by capsaicin. J. Gen. Physiol..

[16] Lishko P.V., Procko E., Jin X., Phelps C.B., Gaudet R. (2007). The ankyrin repeats of TRPV1 bind multiple ligands and modulate channel sensitivity. Neuron.

[17] Jordt S.E., Julius D. (2002). Molecular basis for species-specific sensitivity to “hot” chili peppers. Cell.

[18] Yang F., Xiao X., Cheng W., Yang W., Yu P., Song Z., Yarov-Yarovoy V., Zheng J. (2015). Structural mechanism underlying capsaicin binding and activation of the TRPV1 ion channel. Nat. Chem. Biol..

[19] Jordt S.E., Tominaga M., Julius D. (2000). Acid potentiation of the capsaicin receptor determined by a key extracellular site. Proc. Natl. Acad. Sci. USA.

[20] Yuan P. (2019). Structural biology of thermoTRPV channels. Cell Calcium.

[21] Woo D.H., Jung S.J., Zhu M.H., Park C.K., Kim Y.H., Oh S.B., Lee C.J. (2008). Direct activation of transient receptor potential vanilloid 1(TRPV1) by diacylglycerol (DAG). Mol. Pain.

[22] Wen H., Östman J., Bubb K.J., Panayiotou C., Priestley J.V., Baker M.D., Ahluwalia A. (2012). 20-hydroxyeicosatetraenoic acid (20-HETE) is a novel activator of transient receptor potential vanilloid 1 (TRPV1) channel. J. Biol. Chem..

[23] Smart D., Gunthorpe M.J., Jerman J.C., Nasir S., Gray J., Muir A.I., Chambers J.K., Randall A.D., Davis J.B. (2000). The endogenous lipid anandamide is a full agonist at the human vanilloid receptor (hVR1). Br. J. Pharmacol..

[24] Movahed P., Jönsson B.A.G., Birnir B., Wingstrand J.A., Jørgensen T.D., Ermund A., Sterner O., Zygmunt P.M., Högestätt E.D. (2005). Endogenous unsaturated C18 N-acylethanolamines are vanilloid receptor (TRPV1) agonists. J. Biol. Chem..

[25] Chu C.J., Huang S.M., De Petrocellis L., Bisogno T., Ewing S.A., Miller J.D., Zipkin R.E., Daddario N., Appendino G., Di Marzo V. (2003). N-oleoyldopamine, a novel endogenous capsaicin-like lipid that produces hyperalgesia. J. Biol. Chem..

[26] Yoshida T., Inoue R., Morii T., Takahashi N., Yamamoto S., Hara Y., Tominaga M., Shimizu S., Sato Y., Mori Y. (2006). Nitric oxide activates TRP channels by cysteine S-nitrosylation. Nat. Chem. Biol..

[27] Yang F., Ma L., Cao X., Wang K., Zheng J. (2014). Divalent cations activate TRPV1 through promoting conformational change of the extracellular region. J. Gen. Physiol..

[28] Jara-Oseguera A., Bae C., Swartz K.J. (2016). An external sodium ion binding site controls allosteric gating in TRPV1 channels. eLife.

[29] Patacchini R., Santicioli P., Giuliani S., Maggi C.A. (2005). Pharmacological investigation of hydrogen sulfide (H2S) contractile activity in rat detrusor muscle. Eur. J. Pharmacol..

[30] Bohlen C.J., Priel A., Zhou S., King D., Siemens J., Julius D. (2010). A bivalent tarantula toxin activates the capsaicin receptor, TRPV1, by targeting the outer pore domain. Cell.

[31] Nieto-Posadas A., Picazo-Juárez G., Llorente I., Jara-Oseguera A., Morales-Lázaro S., Escalante-Alcalde D., Islas L.D., Rosenbaum T. (2011). Lysophosphatidic acid directly activates TRPV1 through a C-terminal binding site. Nat. Chem. Biol..

[32] Moiseenkova-Bell V.Y., Stanciu L.A., Serysheva I.I., Tobe B.J., Wensel T.G. (2008). Structure of TRPV1 channel revealed by electron cryomicroscopy. Proc. Natl. Acad. Sci. USA.

[33] De-la-Rosa V., Rangel-Yescas G.E., Ladrón-de-Guevara E., Rosenbaum T., Islas L.D. (2013). Coarse architecture of the transient receptor potential vanilloid 1 (TRPV1) ion channel determined by fluorescence resonance energy transfer. J. Biol. Chem..

[34] Salazar H., Jara-Oseguera A., Hernández-García E., Llorente I., Arias-Olguín I.I., Soriano-García M., Islas L.D., Rosenbaum T. (2009). Structural determinants of gating in the TRPV1 channel. Nat. Struct. Mol. Biol..

[35] Steinberg X., Kasimova M.A., Cabezas-Bratesco D., Galpin J.D., Ladron-de-Guevara E., Villa F., Carnevale V., Islas L., Ahern C.A., Brauchi S.E. (2017). Conformational dynamics in TRPV1 channels reported by an encoded coumarin amino acid. eLife.

[36] Winter J., Walpole C.S.J., Bevan S., James I.F. (1993). Characterization of resiniferatoxin binding sites on sensory neurons: Co-regulation of resiniferatoxin binding and capsaicin sensitivity in adult rat dorsal root ganglia. Neuroscience.

[37] Szallasi A., Blumberg P.M. (1989). Resiniferatoxin, a phorbol-related diterpene, acts as an ultrapotent analog of capsaicin, the irritant constituent in red pepper. Neuroscience.

[38] Yang F., Zheng J. (2017). Understand spiciness: Mechanism of TRPV1 channel activation by capsaicin. Protein Cell.

[39] Bae C., Anselmi C., Kalia J., Jara-Oseguera A., Schwieters C.D., Krepkiy D., Won Lee C., Kim E.H., Kim J.I., Faraldo-Gómez J.D. (2016). Structural insights into the mechanism of activation of the TRPV1 channel by a membrane-bound tarantula toxin. eLife.

[40] Geron M., Kumar R., Zhou W., Faraldo-Gómez J.D., Vásquez V., Priel A. (2018). TRPV1 pore turret dictates distinct DkTx and capsaicin gating. Proc. Natl. Acad. Sci. USA.

[41] Gao Y., Cao E., Julius D., Cheng Y. (2016). TRPV1 structures in nanodiscs reveal mechanisms of ligand and lipid action. Nature.

[42] Gavva N.R., Klionsky L., Qu Y., Shi L., Tamir R., Edenson S., Zhang T.J., Viswanadhan V.N., Toth A., Pearce L.V. (2004). Molecular determinants of vanilloid sensitivity in TRPV1. J. Biol. Chem..

[43] Phillips E., Reeve A., Bevan S., McIntyre P. (2004). Identification of species-specific determinants of the action of the antagonist capsazepine and the agonist PPAHV on TRPV1. J. Biol. Chem..

[44] Cao E. (2020). Structural mechanisms of transient receptor potential ion channels. J. Gen. Physiol..

[45] Liu T.Y., Chu Y., Mei H.R., Chang D., Chuang H.H. (2020). Two vanilloid ligand bindings per channel are required to transduce capsaicin-activating stimuli. Front. Mol. Neurosci..

[46] Méndez-Durán A., Ignorosa-Luna M.H., Pérez-Aguilar G., Rivera-Rodríguez F.J., González-Izquierdo J.J., Dávila-Torres J. (2016). Current status of alternative therapies renal function at the Instituto Mexicano del Seguro Social. Rev. Med. Inst. Mex Seguro Soc..

[47] Voss A., Reinhart M., Sprecher H. (1992). Differences in the interconversion between 20- and 22-carbon (n-3) and (n-6) polyunsaturated fatty acids in rat liver. Biochim. Biophys. Acta.

[48] Matta J.A., Miyares R.L., Ahern G.P. (2007). TRPV1 is a novel target for omega-3 polyunsaturated fatty acids. J. Physiol. (Lond.).

[49] Roman R.J. (2002). P-450 metabolites of arachidonic acid in the control of cardiovascular function. Physiol. Rev..

[50] Hwang S.H., Wagner K., Xu J., Yang J., Li X., Cao Z., Morisseau C., Lee K.S.S., Hammock B.D. (2017). Chemical synthesis and biological evaluation of ω-hydroxy polyunsaturated fatty acids. Bioorg. Med. Chem. Lett..

[51] Patwardhan A.M., Scotland P.E., Akopian A.N., Hargreaves K.M. (2009). Activation of TRPV1 in the spinal cord by oxidized linoleic acid metabolites contributes to inflammatory hyperalgesia. Proc. Natl. Acad. Sci. USA.

[52] Patwardhan A.M., Akopian A.N., Ruparel N.B., Diogenes A., Weintraub S.T., Uhlson C., Murphy R.C., Hargreaves K.M. (2010). Heat generates oxidized linoleic acid metabolites that activate TRPV1 and produce pain in rodents. J. Clin. Investig..

[53] Hwang S.W., Cho H., Kwak J., Lee S.Y., Kang C.J., Jung J., Cho S., Min K.H., Suh Y.G., Kim D. (2000). Direct activation of capsaicin receptors by products of lipoxygenases: Endogenous capsaicin-like substances. Proc. Natl. Acad. Sci. USA.

[54] Story G.M., Peier A.M., Reeve A.J., Eid S.R., Mosbacher J., Hricik T.R., Earley T.J., Hergarden A.C., Andersson D.A., Hwang S.W. (2003). ANKTM1, a TRP-like channel expressed in nociceptive neurons, is activated by cold temperatures. Cell.

[55] Gregus A.M., Doolen S., Dumlao D.S., Buczynski M.W., Takasusuki T., Fitzsimmons B.L., Hua X.Y., Taylor B.K., Dennis E.A., Yaksh T.L. (2012). Spinal 12-lipoxygenase-derived hepoxilin A3 contributes to inflammatory hyperalgesia via activation of TRPV1 and TRPA1 receptors. Proc. Natl. Acad. Sci. USA.

[56] Bhave G., Hu H.J., Glauner K.S., Zhu W., Wang H., Brasier D.J., Oxford G.S., Gereau R.W. (2003). Protein kinase C phosphorylation sensitizes but does not activate the capsaicin receptor transient receptor potential vanilloid 1 (TRPV1). Proc. Natl. Acad. Sci. USA.

[57] Numazaki M., Tominaga T., Toyooka H., Tominaga M. (2002). Direct phosphorylation of capsaicin receptor VR1 by protein kinase C epsilon and identification of two target serine residues. J. Biol. Chem..

[58] Alsalem M., Wong A., Millns P., Arya P.H., Chan M.S.L., Bennett A., Barrett D.A., Chapman V., Kendall D.A. (2013). The contribution of the endogenous TRPV1 ligands 9-HODE and 13-HODE to nociceptive processing and their role in peripheral inflammatory pain mechanisms. Br. J. Pharmacol..

[59] Genicot S.M., Groisillier A., Rogniaux H., Meslet-Cladiére L., Barbeyron T., Helbert W. (2014). Discovery of a novel iota carrageenan sulfatase isolated from the marine bacterium Pseudoalteromonas carrageenovora. Front. Chem..

[60] Ziboh V.A., Miller C.C., Cho Y. (2000). Metabolism of polyunsaturated fatty acids by skin epidermal enzymes: Generation of antiinflammatory and antiproliferative metabolites. Am. J. Clin. Nutr..

[61] Green D., Ruparel S., Gao X., Ruparel N., Patil M., Akopian A., Hargreaves K. (2016). Central activation of TRPV1 and TRPA1 by novel endogenous agonists contributes to mechanical allodynia and thermal hyperalgesia after burn injury. Mol. Pain.

[62] McKemy D.D., Liedtke W.B., Heller S. (2007). TRPM8: The cold and menthol receptor. TRP Ion Channel Function in Sensory Transduction and Cellular Signaling Cascades.

[63] Battista N., Di Tommaso M., Bari M., Maccarrone M. (2012). The endocannabinoid system: An overview. Front. Behav. Neurosci..

[64] Iannotti F.A., Di Marzo V., Petrosino S. (2016). Endocannabinoids and endocannabinoid-related mediators: Targets, metabolism and role in neurological disorders. Prog. Lipid Res..

[65] Leishman E., Bradshaw H.B. (2015). N-acyl amides: Ubiquitous endogenous cannabimimetic lipids that are in the right place at the right time. The Endocannabinoidome.

[66] Raboune S., Stuart J.M., Leishman E., Takacs S.M., Rhodes B., Basnet A., Jameyfield E., McHugh D., Widlanski T., Bradshaw H.B. (2014). Novel endogenous N-acyl amides activate TRPV1-4 receptors, BV-2 microglia, and are regulated in brain in an acute model of inflammation. Front. Cell. Neurosci..

[67] Okamoto Y., Morishita J., Tsuboi K., Tonai T., Ueda N. (2004). Molecular characterization of a phospholipase D generating anandamide and its congeners. J. Biol. Chem..

[68] Blancaflor E.B., Kilaru A., Keereetaweep J., Khan B.R., Faure L., Chapman K.D. (2014). N-acylethanolamines: Lipid metabolites with functions in plant growth and development. Plant J..

[69] Zygmunt P.M., Petersson J., Andersson D.A., Chuang H., Sørgård M., Di Marzo V., Julius D., Högestätt E.D. (1999). Vanilloid receptors on sensory nerves mediate the vasodilator action of anandamide. Nature.

[70] Marzo V.D., Bisogno T., De Petrocellis L. (2001). Endocannabinoids part II: Pathological CNS conditions involving the endocannabinoid system and their possible treatment with endocannabinoid-based drugs. Expert Opin. Ther. Targets.

[71] Bisogno T., Maurelli S., Melck D., De Petrocellis L., Di Marzo V. (1997). Biosynthesis, uptake, and degradation of anandamide and palmitoylethanolamide in leukocytes. J. Biol. Chem..

[72] Wang X., Miyares R.L., Ahern G.P. (2005). Oleoylethanolamide excites vagal sensory neurones, induces visceral pain and reduces short-term food intake in mice via capsaicin receptor TRPV1. J. Physiol. (Lond.).

[73] Almási R., Szoke E., Bölcskei K., Varga A., Riedl Z., Sándor Z., Szolcsányi J., Petho G. (2008). Actions of 3-methyl-N-oleoyldopamine, 4-methyl-N-oleoyldopamine and N-oleoylethanolamide on the rat TRPV1 receptor in vitro and in vivo. Life Sci..

[74] Kuehl F.A., Jacob T.A., Ganley O.H., Ormond R.E., Meisinger M.A.P. (1957). The identification of N-(2-hydroxyethyl)-palmitamide as a naturally occurring anti-inflammatory agent. J. Am. Chem. Soc..

[75] Re G., Barbero R., Miolo A., Di Marzo V. (2007). Palmitoylethanolamide, endocannabinoids and related cannabimimetic compounds in protection against tissue inflammation and pain: Potential use in companion animals. Vet. J..

[76] Petrosino S., Schiano Moriello A., Cerrato S., Fusco M., Puigdemont A., De Petrocellis L., Di Marzo V. (2016). The anti-inflammatory mediator palmitoylethanolamide enhances the levels of 2-arachidonoyl-glycerol and potentiates its actions at TRPV1 cation channels. Br. J. Pharmacol..

[77] Lo Verme J., Fu J., Astarita G., La Rana G., Russo R., Calignano A., Piomelli D. (2005). The nuclear receptor peroxisome proliferator-activated receptor-alpha mediates the anti-inflammatory actions of palmitoylethanolamide. Mol. Pharmacol..

[78] Petrosino S., Iuvone T., Di Marzo V. (2010). N-palmitoyl-ethanolamine: Biochemistry and new therapeutic opportunities. Biochimie.

[79] Costa B., Comelli F., Bettoni I., Colleoni M., Giagnoni G. (2008). The endogenous fatty acid amide, palmitoylethanolamide, has anti-allodynic and anti-hyperalgesic effects in a murine model of neuropathic pain: Involvement of CB(1), TRPV1 and PPARgamma receptors and neurotrophic factors. Pain.

[80] Ho W.S.V., Barrett D.A., Randall M.D. (2008). “Entourage” effects of N-palmitoylethanolamide and N-oleoylethanolamide on vasorelaxation to anandamide occur through TRPV1 receptors. Br. J. Pharmacol..

[81] Gunthorpe M.J., Rami H.K., Jerman J.C., Smart D., Gill C.H., Soffin E.M., Luis Hannan S., Lappin S.C., Egerton J., Smith G.D. (2004). Identification and characterisation of SB-366791, a potent and selective vanilloid receptor (VR1/TRPV1) antagonist. Neuropharmacology.

[82] Ambrosino P., Soldovieri M.V., Russo C., Taglialatela M. (2013). Activation and desensitization of TRPV1 channels in sensory neurons by the PPARα agonist palmitoylethanolamide. Br. J. Pharmacol..

[83] Déciga-Campos M., Ramírez-Marín P.M., López-Muñoz F.J. (2015). Synergistic antinociceptive interaction between palmitoylethanolamide and tramadol in the mouse formalin test. Eur. J. Pharmacol..

[84] Connor M., Vaughan C.W., Vandenberg R.J. (2010). N-acyl amino acids and N-acyl neurotransmitter conjugates: Neuromodulators and probes for new drug targets. Br. J. Pharmacol..

[85] Zhang M., Ruwe D., Saffari R., Kravchenko M., Zhang W. (2020). Effects of TRPV1 activation by capsaicin and endogenous N-arachidonoyl taurine on synaptic transmission in the prefrontal cortex. Front. Neurosci..

[86] Yang K., Kumamoto E., Furue H., Yoshimura M. (1998). Capsaicin facilitates excitatory but not inhibitory synaptic transmission in substantia gelatinosa of the rat spinal cord. Neurosci. Lett..

[87] Marinelli S., Vaughan C.W., Christie M.J., Connor M. (2002). Capsaicin activation of glutamatergic synaptic transmission in the rat locus coeruleus in vitro. J. Physiol. (Lond.).

[88] Saghatelian A., McKinney M.K., Bandell M., Patapoutian A., Cravatt B.F. (2006). A FAAH-regulated class of *N-* acyl taurines that activates TRP ion channels. Biochemistry.

[89] Sawchenko P.E., Swanson L.W. (1983). The organization of forebrain afferents to the paraventricular and supraoptic nuclei of the rat. J. Comp. Neurol..

[90] Sue Carter C. (2018). Oxytocin and human evolution. Curr. Top. Behav. Neurosci..

[91] Nersesyan Y., Demirkhanyan L., Cabezas-Bratesco D., Oakes V., Kusuda R., Dawson T., Sun X., Cao C., Cohen A.M., Chelluboina B. (2017). Oxytocin modulates nociception as an agonist of pain-sensing TRPV1. Cell Rep..

[92] Trevisani M., Patacchini R., Nicoletti P., Gatti R., Gazzieri D., Lissi N., Zagli G., Creminon C., Geppetti P., Harrison S. (2005). Hydrogen sulfide causes vanilloid receptor 1-mediated neurogenic inflammation in the airways. Br. J. Pharmacol..

[93] Schicho R., Krueger D., Zeller F., Von Weyhern C.W.H., Frieling T., Kimura H., Ishii I., De Giorgio R., Campi B., Schemann M. (2006). Hydrogen sulfide is a novel prosecretory neuromodulator in the Guinea-pig and human colon. Gastroenterology.

[94] Lu W., Li J., Gong L., Xu X., Han T., Ye Y., Che T., Luo Y., Li J., Zhan R. (2014). H_2_S modulates duodenal motility in male rats via activating TRPV1 and K(ATP) channels. Br. J. Pharmacol..

[95] Sun H.Z., Gong X.Y., Wu L., Wang X.X., Nie Y.N., Shang R., Wang H., Li Y.C., Sun Q.F., Gao P.F. (2018). Hydrogen sulfide modulates gastric acid secretion in rats via involvement of substance P and nuclear factor-κB signaling. J. Physiol. Pharmacol..

[96] Holzer P., Holzer-Petsche U. (2001). Tachykinin receptors in the gut: Physiological and pathological implications. Curr. Opin. Pharmacol..

[97] Graefe S., Mohiuddin S.S. (2020). Biochemistry, substance P. StatPearls.

[98] Ohsawa M., Miyabe Y., Katsu H., Yamamoto S., Ono H. (2013). Identification of the sensory nerve fiber responsible for lysophosphatidic acid-induced allodynia in mice. Neuroscience.

[99] Ueda H., Matsunaga H., Olaposi O.I., Nagai J. (2013). Lysophosphatidic acid: Chemical signature of neuropathic pain. Biochim. Biophys. Acta.

[100] Inoue M., Ma L., Aoki J., Chun J., Ueda H. (2008). Autotaxin, a synthetic enzyme of lysophosphatidic acid (LPA), mediates the induction of nerve-injured neuropathic pain. Mol. Pain.

[101] Inoue M., Rashid M.H., Fujita R., Contos J.J.A., Chun J., Ueda H. (2004). Initiation of neuropathic pain requires lysophosphatidic acid receptor signaling. Nat. Med..

[102] Chemin J., Patel A., Duprat F., Zanzouri M., Lazdunski M., Honoré E. (2005). Lysophosphatidic acid-operated K^+^ channels. J. Biol. Chem..

[103] Stirling L., Williams M.R., Morielli A.D. (2009). Dual roles for RHOA/RHO-kinase in the regulated trafficking of a voltage-sensitive potassium channel. Mol. Biol. Cell.

[104] Kittaka H., Uchida K., Fukuta N., Tominaga M. (2017). Lysophosphatidic acid-induced itch is mediated by signalling of LPA5 receptor, phospholipase D and TRPA1/TRPV1. J. Physiol. (Lond.).

[105] Czirják G., Tóth Z.E., Enyedi P. (2004). The two-pore domain K^+^ channel, TRESK, is activated by the cytoplasmic calcium signal through calcineurin. J. Biol. Chem..

[106] Telezhkin V., Reilly J.M., Thomas A.M., Tinker A., Brown D.A. (2012). Structural requirements of membrane phospholipids for M-type potassium channel activation and binding. J. Biol. Chem..

[107] Jang Y., Lee M.H., Lee J., Jung J., Lee S.H., Yang D.J., Kim B.W., Son H., Lee B., Chang S. (2014). TRPM2 mediates the lysophosphatidic acid-induced neurite retraction in the developing brain. Pflugers Arch..

[108] Cao E., Cordero-Morales J.F., Liu B., Qin F., Julius D. (2013). TRPV1 channels are intrinsically heat sensitive and negatively regulated by phosphoinositide lipids. Neuron.

[109] Morales-Lázaro S.L., Serrano-Flores B., Llorente I., Hernández-García E., González-Ramírez R., Banerjee S., Miller D., Gududuru V., Fells J., Norman D. (2014). Structural determinants of the transient receptor potential 1 (TRPV1) channel activation by phospholipid analogs. J. Biol. Chem..

[110] Canul-Sánchez J.A., Hernández-Araiza I., Hernández-García E., Llorente I., Morales-Lázaro S.L., Islas L.D., Rosenbaum T. (2018). Different agonists induce distinct single-channel conductance states in TRPV1 channels. J. Gen. Physiol..

[111] Chung M.K., Güler A.D., Caterina M.J. (2008). TRPV1 shows dynamic ionic selectivity during agonist stimulation. Nat. Neurosci..

